# Compliance of Bhabhatron‐II telecobalt unit with IEC standard ‐ radiation safety

**DOI:** 10.1120/jacmp.v10i2.2963

**Published:** 2009-04-28

**Authors:** G Sahani, Munish Kumar, PK Dash Sharma, DN Sharma, Kanta Chhokra, Bibekananda Mishra, SP Agarwal, RK Kher

**Affiliations:** ^1^ Radiological Safety Division Atomic Energy Regulatory Board Mumbai‐ 94 India; ^2^ Radiological Physics & Advisory Division Bhabha Atomic Research Centre Mumbai‐85 India; ^3^ Radiation Safety & Systems Division Bhabha Atomic Research Centre Mumbai‐85 India; ^4^ Homi Bhabha National Institute (Deemed University) Mumbai‐94 India

**Keywords:** IEC, radiation safety standards, telecobalt, dosimeters

## Abstract

Bhabha Atomic Research Centre, Mumbai, India designed and developed a telecobalt unit, Bhabhatron‐II. In this paper, the results pertaining to radiation safety of the indigenously developed Bhabhatron‐II telecobalt unit are reported. The various tests were carried out as per requirements of International Electrotechnical Commission standards and acceptance criteria. Various devices such as ${\text{CaSO}}_{4}$: Dy‐based thermoluminescent dosimeters, Farmer‐type ionization chamber, water phantom, and radiographic films were used. All the parameters pertaining to radiation leakage/transmission were within the tolerance limits, as per the IEC standard 60601–2–11, except the collimator transmission through X collimators (upper jaw), which marginally exceeds the tolerance limit.

PACS number: 87.52Tr, 87.56By, 87.56.FC

## I. INTRODUCTION

Telecobalt units are widely used in developing countries for cancer treatment and are preferred over medical linear accelerators (linacs) because of: i) low cost, ii) low maintenance cost, iii) lower power requirements, and iv) less down time. It is recognized that medical linac has some advantages over telecobalt machines such as variable dose rates, multiple photon and electron beams and energies, and smaller beam penumbra. At present in India, there are only 399 teletherapy units (280 telecobalt units and 119 linacs). On the basis of data pertaining to cancer incidence in India, it is estimated that more than 1000 teletherapy units will be required in the near future. It is worth mentioning that out of the 280 telecobalt units, almost all are imported. Further, the imported telecobalt units are quite expensive, which is a major hindrance for establishing radiotherapy centers in rural India. In view of the above, Bhabha Atomic Research Centre, Mumbai, India has designed and developed a prototype telecobalt unit, which has been named Bhabhatron‐I.^(^ ^1^ ^–^ ^2^ ^)^ After receiving feedback on the operations of this unit, a modified model was developed, Bhabhatron‐II, and has a maximum source capacity of 555 TBq of Co60. The technical details of Bhabhatron‐II are listed in Table 1. Telecobalt machines have become obsolete in developed countries and no efforts are being made for further advancement. However, there is a huge demand of telecobalt units in developing countries like India because of the reasons mentioned above. In India, efforts are also in progress for upgrading telecobalt units with advanced technology such as multileaf collimators (MLCs).^(^ ^3^ ^–^ ^4^ ^)^ The secondary collimators of the Bhabhatron‐II are of block type; therefore there is the possibility of upgrading the units with multileaf collimators by replacing either pair of jaws.

**Table 1:** Technical specifications of Bhabhatron‐II telecobalt unit.

*Sr. No*.	*Parameters*	*Specified Value*
1.	Maximum source strength capacity	555 TBq (250 RMM) of C60
2.	Source to axis distance (SAD)	80 cm
3.	Maximum field size at isocenter	35 cm×35 cm
4.	^a^Minimum field size at isocenter	~0 cm×0 cm
5.	Optical distance indicator (ODI) scale range	SAD±20 cm
6.	Gantry	Motorized, isocentric motion, rotation: ±180°
7.	Collimator	Motorized, ±90°
8.	Automatic collimator closure	Standard
9.	Patient positioning table	Motorized: longitudinal, lateral, vertical and rotational motions
10.	Wedge filters	A set of manual wedge filters of various wedge angles from 15°, 30°, 45° and 60° are provided
11.	Provision for wedge filter interlock	Provided
12.	Display of set parameters inside the room	TFT monitor provided

a Jaws are can be closed to 0 cm×0 cm physically; however inter‐jaw transmission (radiation) is observed by exposing the therapy verification film. The major difference between the Bhabhatron‐II unit and a commercially available telecobalt unit is in secondary collimator design. The design of secondary collimators of the Bhabhatron‐II telecobalt unit is block type, whereas those of commercially available telecobalt units are multi‐vein type.

In India, the Atomic Energy Regulatory Board (AERB) ensures that the unit complies with national/international standards before granting type approval certificate to the manufacturer. It is mandatory that, before the unit is used for clinical applications, it shall comply with various standards such as electrical, mechanical, dosimetric, and radiation safety. In this paper, the results pertaining only to radiation safety of the Bhabhatron‐II telecobalt unit are reported. Other tests such as electrical, mechanical and dosimetric are beyond the scope of this paper. The radiation leakage measurements with beam ON/OFF and during source transition were performed following the requirements of the International Electrotechnical Commission,^(5)^ and national requirements as stated in RPAD/telegamma/QA/01.^(6)^ To obtain the measurements, four different dosimetric devices were used: thermoluminescent dosimeters (discs and powder), 0.65 cc Farmer‐type ionization chamber (Scanditronix, model FC65), and radiographic films. The results of the study were useful not only for the manufacturer in modifying the telecobalt unit but they also helped in ensuring compliance with the prevailing standards.

Following the IEC 60601–2–11 requirements, the conditions noted below shall be satisfied as far as radiation leakage/transmission is concerned.^(5)^

### A. Protection of the patient against radiation outside the radiation beam

#### A.1 Leakage radiation through beam limiting devices (secondary collimator) during irradiation

In the case of beam ON position, beam limiting devices are used to attenuate the radiation so that the absorbed dose at the normal treatment distance (NTD) anywhere in the area protected by the beam limiting device shall not exceed 2% of the maximum absorbed dose for a 10 cm×10 cm radiation field measured on the radiation beam axis at the same distance. For equipment in which the maximum field size of the radiation beam exceeds 500 cm^2^ at NTD, the following additional limits shall be applied for collimator transmission.

For square fields of any size, the product of the average absorbed dose due to leakage radiation through the beam limiting devices and the maximum area able to be protected by the beam limiting devices shall not exceed one‐tenth of the product of the maximum absorbed dose on the radiation beam axis and the area of the radiation beam for a field size of 10 cm×10 cm.

If M is the maximum area able to be protected by the beam limiting devices (here secondary collimator) in cm^2^ and DL is the average absorbed dose due to the leakage radiation through the beam limiting devices then:

(1) $$\begin{array}{rl} & DL\times M<1/10\times maximum\;absorbed\;dose\;for\;10\;cm\times 10\;cm\\ & field\;size\times area\;for\;10\;cm\times 10\;cm \end{array}$$

All the values of absorbed dose and area are referred at NTD.

#### A.2 Leakage radiation outside the maximum radiation beam

The telecobalt head shall be provided with protective shielding which shall attenuate the radiation while the source is in transition or in the fully ON position, sufficient to satisfy the following conditions:

i) In a plane circular surface of radius 2 m centered on and normal to the radiation beam axis at NTD and outside the area of the maximum radiation beam area, the absorbed dose rate due to leakage radiation shall not exceed a maximum of 0.2% and an average of 0.1% of the maximum absorbed dose rate measured at the point of intersection of the radiation beam axis and the plane surface for a 10 cm×10 cm field.
ii) The absorbed dose rate due to leakage radiation measured at a distance of 1 m from the radiation source shall not exceed 0.5% of the maximum absorbed dose rate on the radiation beam axis measured at a distance of 1 m from the radiation source.

### B. radiation safety for persons other than patients

#### B.1 Stray radiation in the beam off position

The protective shielding shall attenuate the radiation such that with the beam control mechanism in the beam OFF position, the absorbed dose rate due to stray radiation (including radiation from radioactive material other that the radiation source) measured at a distance of 1 m from the radiation source shall not exceed 20 μGy/hr. Measurements shall be the average value obtained over a surface area $\leq 100\;{\text{cm}}^{2}$. Also at any readily accessible position, 5 cm from the surface of the protective shielding, the absorbed dose rate due to stray radiation shall not exceed 200 μGy/hr and the measurements shall be the average value obtained over a surface of area $\leq 10\;{\text{cm}}^{2}$.

### C. relative surface dose

The relative absorbed dose at 0.5 mm depth on the radiation beam axis with normal treatment distance of 80 cm (for Bhabhatron‐II telecobalt) shall not exceed: i) 70% of absorbed dose at 5 mm depth for 10 cm×10 cm radiation field size, and ii) 90% of absorbed dose at 5 mm depth for maximum radiation field size available (i.e. 35 cm×35 cm).

## II. MATERIALS AND METHODS

For evaluation of radiation leakage and relative surface dose measurements, the dose rate of the machine at depth of maximum dose for various field sizes is required. Dose rate was measured using calibrated Farmer‐type ionization chamber (FC65) with electrometer supplied by Scanditronix in water phantom 30 cm×30 cm×30 cm. To find the point of maximum radiation leakage, radiographic films were used in beam ON and OFF conditions.

### A. Evaluation of collimator transmission

A radiographic film was placed in the plane normal to the radiation beam axis at the normal treatment distance by setting X collimators for minimum field size and Y collimators for maximum field size so that transmission occurred only through the pair of X collimators. Similarly another radiographic film was exposed by setting Y collimator for minimum field size and X collimator for maximum field size. The exposed radiographic films were evaluated to locate the point of maximum leakage radiation through the X and Y collimators in source ON condition. For the measurement of absorbed dose due to collimator transmission (radiation leakage), ${\text{CaSO}}_{4}$: Dy (0.05 mole %) teflon embedded thermoluminescent (TL) discs^(7)^ (having a thickness of 0.8 mm and a diameter of 13.3 mm) with 5 mm buildup of virtual water were used at the point of maximum – as well as uniform – radiation leakage points.

**Figure 1:**
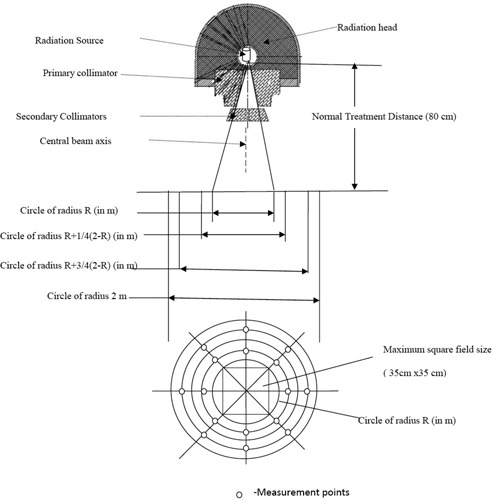
Radiation leakage measurement at 16 points in the plane of radius 2 m centered at isocenter and normal to the central beam axis.

As the maximum square field size of radiation for this machine is 35 cm×35 cm, which is more than 500 cm^2^, an additional condition of collimator transmission measurement must be applied. In this case, for the measurement of average absorbed dose due to leakage radiation through the beam limiting devices, 12 points were chosen. These points were: four points located on the two major axes at a distance of 1/3 R from the radiation beam axis, and eight points located on the two major axes and on the two diagonals at a distance of 2/3 R from the radiation beam axis. All values of the absorbed dose and area are referred to the NTD (see Fig. 1). The parameter R is ${\left\{{\left(17.5/2\right)}^{2}+{\left(17.5/2\right)}^{2}\right\}}^{1/2}=24.75\;\text{cm}$, which is the radius of the circle covering the maximum definable square field size (i.e. 35 cm×35 cm). The TL dosimeters were placed with 5 mm buildup of virtual water. All values of the absorbed dose and area are referred to NTD.

### B. radiation leakage measurements in the patient plane

A circular plane of radius 2 m centered on and normal to the central radiation beam axis at the normal treatment distance (NTD) and outside the area of the maximum radiation beam is called patient plane.^(8)^ ${\text{CaSO}}_{4}$: Dy TL discs were placed with 5 mm buildup of virtual water at the 16 test points as defined in Fig. 1 for radiation leakage measurement in the patient plane.

### C. Measurement of the radiation leakage in other than patient plane

**Figure 2:**
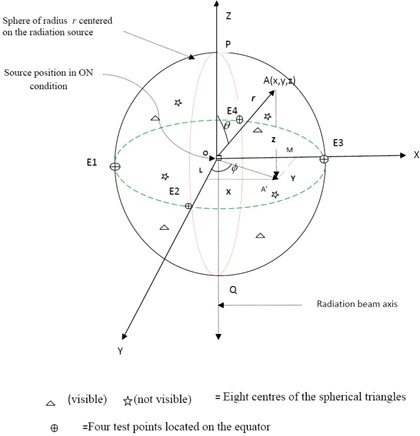
Location of 13 points for radiation head leakage measurement in source ON condition.

Following IEC‐60601‐2‐11 requirements, it is tedious to locate the 13 test points distributed evenly on the sphere of radius r (here r=1 m) with source at the center of the sphere as explained in Fig. 2. However, these points can be easily located if the coordinates of the points measurement are known in terms of Cartesian co‐ordinates. As Fig. 2, we have three great circles – E1–E2–E3–E4, P‐E2‐Q‐E4, and P‐E3‐Q‐E1 – which are perpendicular to each other and pass through the center of the sphere. Points P and Q are the poles of the sphere. PQ is the central axis of the radiation beam. The equator is equally divided into four points by the great circles P‐E2‐Q‐E4 and P‐E3‐Q‐E1 passing through poles P and Q. This creates eight identical spherical triangles. If A (x, y, z) can be any point on the spherical triangle E2–E3‐P and A′ can be its projection on the XY plane (equator plane), then we can have the following relations between polar and Cartesian coordinates:

(2) x=rsin⁡θsin⁡ϕ,y=rsin⁡θcos⁡ϕ,and z=rcos⁡θ

where θ=angle between radius OA (r) and Z axis, φ=angle between OA′ and Y axis

Spherical coordinates for the center of spherical triangle E2‐E3‐P are; r=1 m,θ=45°, and φ=45°. Using above relation (Eq. 2), the values of the Cartesian coordinates for the center of spherical triangle E2‐E3‐P will be:

(3) $$\begin{array}{rl} & x=1\sin45^{\circ }\sin45^{\circ }m=0.5\;m,\;y=1\sin45^{\circ }\cos45^{\circ }m=0.5\;m,\\ & z=1\cos45^{\circ }m=0.707\;m \end{array}$$

Similarly, one can also locate the centers of the other seven spherical triangles. The TL discs were irradiated with 5 mm buildup of virtual water at the above test points, including 4 test points El, E2, E3, E4 on the equator and one on the pole *P*.

### D. Leakage radiation from the source head in source OFF condition

Radiographic films were wrapped around the unit head to locate the point of maximum leakage radiation in the source OFF condition (source parking position). For the measurement of absorbed dose due to radiation leakage through the head, TL discs with 5 mm buildup of virtual water were used at the point of maximum radiation leakage (5 cm from the accessible surface and at 1 m from the source in source OFF position) and irradiated for 36 hours in order to achieve better statistical accuracy of the measurement (as the radiation leakage rate is very low). The linearity of the TL dosimeters was checked up to 5Gy for exposures carried out using Co60 source. It was found that the graph of absorbed dose versus TL output was linear within the ±3%. All the irradiated TL discs were read on a calibrated hot gas TLD reader.^(9)^

### E. Relative surface dose

Following IEC‐60601‐2‐11 requirements, relative surface dose needs to be measured at the water equivalent depth of 0.5 mm. The dose measurement of the dose buildup region is tedious^(^ ^10^ ^–^ ^11^ ^)^ because of the steep dose gradient and, therefore, the dosimeter thickness should be extremely thin. For this purpose, the TL phosphor of average grain size less than 40μ was used. The absorbed dose was measured on the radiation beam axis with a source‐to‐surface distance of 80 cm by placing the ${\text{CaSO}}_{4}$: Dy TL phosphors with 0.5 mm water equivalent buildup. The measurements were carried out using a 30 cm×30 cm×20 cm solid water phantom. The TL phosphor was irradiated for both 10 cm×10 cm irradiation field size and also for maximum irradiation field size (i.e. 35 cm×35 cm). The irradiated phosphor was read on a calibrated TL reader and the reproducibility of measurements was better than ±1%.

## III. RESULTS AND DISCUSSION

### A. Collimator transmission

From Table 2, it is evident that the maximum collimator transmission is 2.1% through the X collimator and 1.5 % through the Y collimator of the maximum absorbed dose NTD for a 10 cm×10 cm field size. Thus we observe that collimator transmission except through one of the X collimators (upper jaw), which marginally exceeds the limit, are within the permissible limit (2%). The high value of the X collimator transmission is probably due to nonuniformity of the material density during manufacturing^†^. Additional tests for collimator transmission shows (see Table 3) the average absorbed dose due to leakage radiation is 0.259 cGy/min and the absorbed dose rate measured for 10 cm×10 cm open radiation field size at depth of maximum dose with SSD 80 cm (source‐to‐surface dustance=80 cm) is 177.10 cGy/min. By using the above values, it has been found that inequality (1) is satisfied.

**Table 2:** Results for collimator transmission in source ON condition.

*Secondary collimator setting*	*Positions of TL discs*	*Average dose rate due to radiation leakage (cGy/min)*	*% Radiation leakage* ^a^
Pair of X jaws completely closed and pair of Y jaws completely open	1	3.7	2.1
	2	2.7	1.5
	3	3.0	1.7
	4	3.7	2.1
Pair of Y jaws completely closed and pair of X jaws completely open	5	2.5	1.4
	6	2.7	1.5
	7	2.5	1.4
	8	2.3	1.3

a Percentage radiation leakage of the maximum absorbed dose rate for a 10 cm×10 cm radiation field measured on the radiation beam axis at source to surface distance 80 cm.

**Table 3:** Radiation leakage measurements for testing inequality (1) ‐ collimator transmission.

*Positions of TL discs*	*Absorbed dose rate due to radiation leakage* $\left(10^{3}\times \mu \text{Gy}/\min\right)$	*% Radiation leakage* ^a^
1	3.07	0.17
2	3.12	0.18
3	2.40	0.14
4	3.45	0.19
5	2.64	0.15
6	2.68	0.15
7	2.73	0.15
8	2.43	0.14
9	2.05	0.12
10	2.33	0.13
11	2.21	0.12
12	1.95	0.11

a Percentage radiation leakage of the maximum absorbed dose rate for a 10 cm×10 cm radiation field measured on the radiation beam axis at source to surface distance 80 cm

### B. Leakage radiation in the patient plane

From Table 4, we find that the maximum radiation leakage is 0.019% and an average radiation leakage is 0.0097% of the maximum absorbed dose at NTD for 10 cm×10 cm radiation field size. Thus the results obtained are well below the permissible limit of a 0.2% maximum and a 0.1% average radiation leakage of the maximum absorbed dose at NTD for 10 cm×10 cm radiation field size.

**Table 4:** Radiation leakage in patient plane for source ON position.

*Positions of TL discs* ^a^	*Absorbed dose rate due to radiation leakage (μGy/min)*	*% radiation leakage of maximum absorbed dose for* 10 cm×10 cm *at NTD*
1	335.6	0.019
2	87.4	0.005
3	322.7	0.018
4	105.8	0.006
5	212.2	0.012
6	60.2	0.003
7	179.8	0.010
8	267.2	0.015
9	62.6	0.004
10	287.9	0.016
11	84.2	0.005
12	223.1	0.013
13	72.8	0.004
14	282.1	0.016
15	59.1	0.003
16	90.1	0.005

a For details see Fig. 1.

### C. radiation leakage in other than patient plane

We observe from Table 5 that the maximum radiation leakage is 0.026% of the maximum absorbed dose at 1 m from the source. The result shows that it is well below the permissible limit 0.5% of the maximum absorbed dose at 1 m from the source.

**Table 5:** Radiation leakage in other than patient plane for source ON position.

*Positions of TL discs* ^a^	*Absorbed dose rate due to radiation leakage (μGy/min)*	*% radiation leakage of maximum absorbed dose at 1 meter*
1	150.85	0.013
2	184.32	0.016
3	58.61	0.005
4	79.96	0.007
5	188.41	0.017
6	241.18	0.022
7	102.03	0.009
8	47.94	0.004
9	242.49	0.022
10	227.73	0.020
11	289.26	0.026
12	253.03	0.023
13	199.96	0.018

a For details see Fig. 2.

### D. Radiation leakage from the source head in source OFF condition

From Table 6, it is seen that the radiation leakage levels at 1 m from the source and at 5 cm from any accessible surface were found to be 18.4 μGy/hr and 132.2 μGy/hr, respectively, for maximum rated capacity of the source head. Thus the values are within the permissible limits of 20 μGy/hr at 1m from the source and 200 μGy/hr at 5 cm from any readily accessible surface. Further, from the design data of the machine, the minimum distance of the test point at 5 cm from the surface is 31.8 cm from the source (OFF position). In Table 6, the leakage radiation level at 1 m from source is observed to be 18.4 μGy/hr whereas the leakage radiation level at 5 cm from the surface is 132.2 μGy/hr. It is worth mentioning that the leakage radiation level at 5 cm from the surface comes out to be 13.4 μGy/hr if inverse square law is applied to the value of the leakage radiation at 1 m. This deviation may arise due to the presence of scattered radiation.

**Table 6:** Results for radiation leakage measurements in source OFF (parking) position.

*Measurement locations*	*Absorbed dose rate due radiation leakage* ^a^ *(μGy/hr)*
5 cm from the surface of source head	132.2
1 m from the source	18.4

a Proportioned for maximum source capacity (555 TBq of Co‐60 source)

### E. relative surface dose

The relative absorbed dose at 0.5 mm depth on the radiation beam axis with normal treatment distance 80 cm for 10 cm×10 cm and 35 cm×35 cm field sizes were found to be 63.6% and 79.2%, respectively. These findings are: i) below 70% of absorbed dose at 5 mm depth for 10 cm×10 cm irradiation field size, and ii) below 90% of absorbed dose at 5 mm depth for the maximum irradiation field size available (i.e. 35 cm×35 cm).

Further, from Tables 4 and 5, it is seen that leakage radiation levels at the different points in the same plane at NTD perpendicular to central axis are different because of the head design leading to different thicknesses of the intervening material (TVLs) between the source and the point of measurements. But the maximum leakage radiation observed is within the permissible limit.

The results of the above study have also served as a guideline for the commissioning of other units of the same model. Similar tests carried out for other units were satisfactory. Furthermore, as previously stated, the majority of the telecobalt units being used in our country are imported and, therefore, require comprehensive tests pertaining to radiation leakage/transmission as well as other tests such as dosimetric, mechanical, and electrical. On comparing the data (see Table 7) of the test results pertaining to radiation safety of the Bhabhatron‐II with the imported units (mostly supplied by Theratronics International Ltd.), we found that the Bhabhatron‐II telecobalt unit is as good as any imported unit. Hence the above study has helped the manufacturer to have confidence in the design and production of the Bhabhatron‐II model on commercial scale.

**Table 7:** Comparison between Bhabhatron‐II and Theratron Equnox‐80 telecobalt units.

	*Observed values*		
*Radiation leakage/transmission*	*Bhabhatron‐II*	*Theratron Equnox‐80*	*Tolerance limit*
Maximum collimator transmission through X‐ Jaws^a^	2.1	1.96	≤2.0
Maximum collimator transmission through Y‐ Jaws^a^	1.5	1.33	≤2.0
Radiation leakage from unit head during source ON condition at NTD in the patient plane^a^	max.=0.019	<0.133	max.≤0.20
	Avg.=0.0097	<0.0678	Avg.≤0.10
Radiation leakage from unit head during source ON condition at 1 m from source in other than patient plane^b^	max.=0.026	<0.02	≤0.50
Head Leakage during source OFF condition at 5 cm from unit head surface (Extrapolated for 555 TBq of C60 source)	132.2 μSv/hr	141.5 μSv/hr	≤200 μSv/hr
Head Leakage during source OFF condition at 1 m from the source (Extrapolated for 555 TBq of C60 source)	18.4 μSv/hr	17.3 μSv/hr	≤20 μSv/hr

a Percentage radiation leakage of the maximum absorbed dose rate for a 10 cm×10 cm radiation field measured on the radiation beam axis at source to surface distance 80 cm.

b Percentage radiation leakage of the maximum absorbed dose rate measured on the radiation beam axis at 1 m distance.

## IV. CONCLUSIONS

From this study, it has been found that:

1. Collimator transmission is within 2% of the maximum absorbed dose at NTD for 10 cm×10 cm radiation field size. The exception is through one (2.1%) of the X collimators (upper jaw), which marginally exceeds the limit (2%) but is still within permissible limits.
2. The maximum radiation leakage in the patient plane is 0.019% (limit 0.2%), and an average radiation leakage is 0.097% (limit 0.1%) of the maximum absorbed dose at NTD for 10 cm×10 cm radiation field size.
3. The maximum radiation leakage was found to be 0.026% (0.5%) of the maximum absorbed dose at 1 m from the source in other than the patient plane.
4. The radiation leakage rate at 1 m from the source and at 5 cm from the any other accessible surface are 18.4 μGy/hr (20 μGy/hr) and 132.2 μGy/hr (200 μGy/hr), respectively, for maximum rated capacity of the source head.
5. The relative absorbed dose at 0.5 mm depth on the radiation beam axis with normal treatment distance 80 cm for 10 cm×10 cm and 35 cm×35 cm radiation field sizes were found to be 63.6% (70%) and 79.2% (90%), respectively.

In view of the above, it is stated that all the radiation leakage/transmission parameters are within the tolerance limit specified by the relevant IEC standard, except the collimator transmission through one of the X collimators (upper jaws) which marginally exceeds the tolerance limit.

The above study was helpful to the regulatory body (AERB) for evaluating and approving the type of unit specified in this paper.

## ACKNOWLEDGEMENTS

Shri S.K. Sharma, Chairman, AERB and Shri H. S. Kushwaha, Director Health, Safety and Environment Group (HS&EG), BARC are the guiding force behind this work and the authors are thankful to them for their encouragement. The author (M. K.) would like to thank Dr. Y. S. Mayya, Head, RP&AD, BARC for encouragement.

## Footnotes

† After review of the above results, comprehensive quality control of the material (used for collimators) during manufacturing was ensured which brought down the transmission through X collimator to 1.5% (maximum value). At present seven Bhabhatron‐II telecobalt units complying the IEC standard (IEC‐60601‐2‐11) and other national requirement are operating successfully at various radiotherapy centers in India.
